# Nicotinamide Riboside Regulates Chemotaxis to Decrease Inflammation and Ameliorate Functional Recovery Following Spinal Cord Injury in Mice

**DOI:** 10.3390/cimb46020082

**Published:** 2024-02-01

**Authors:** Yan Li, Chunjia Zhang, Zihan Li, Fan Bai, Yingli Jing, Han Ke, Shuangyue Zhang, Yitong Yan, Yan Yu

**Affiliations:** 1 Institute of Rehabilitation Medicine, China Rehabilitation Science Institute, China Rehabilitation Research Center, Beijing Key Laboratory of Neural Injury and Rehabilitation, Beijing 100068, China; 2 Center of Neural Injury and Repair, Beijing Institute for Brain Disorders, Beijing 100068, China; 3 School of Rehabilitation, Capital Medical University, Beijing 100068, China

**Keywords:** spinal cord injury, nicotinamide riboside, NAD^+^, neuroinflammation, functional recovery

## Abstract

Changes in intracellular nicotinamide adenine dinucleotide (NAD^+^) levels have been observed in various disease states. A decrease in NAD^+^ levels has been noted following spinal cord injury (SCI). Nicotinamide riboside (NR) serves as the precursor of NAD^+^. Previous research has demonstrated the anti-inflammatory and apoptosis-reducing effects of NR supplements. However, it remains unclear whether NR exerts a similar role in mice after SCI. The objective of this study was to investigate the impact of NR on these changes in a mouse model of SCI. Four groups were considered: (1) non-SCI without NR (Sham), (2) non-SCI with NR (Sham +NR), (3) SCI without NR (SCI), and (4) SCI with NR (SCI + NR). Female C57BL/6J mice aged 6–8 weeks were intraperitoneally administered with 500 mg/kg/day NR for a duration of one week. The supplementation of NR resulted in a significant elevation of NAD^+^ levels in the spinal cord tissue of mice after SCI. In comparison to the SCI group, NR supplementation exhibited regulatory effects on the chemotaxis/recruitment of leukocytes, leading to reduced levels of inflammatory factors such as IL-1β, TNF-α, and IL-22 in the injured area. Moreover, NR supplementation notably enhanced the survival of neurons and synapses within the injured area, ultimately resulting in improved motor functions after SCI. Therefore, our research findings demonstrated that NR supplementation had inhibitory effects on leukocyte chemotaxis, anti-inflammatory effects, and could significantly improve the immune micro-environment after SCI, thereby promoting neuronal survival and ultimately enhancing the recovery of motor functions after SCI. NR supplementation showed promise as a potential clinical treatment strategy for SCI.

## 1. Introduction

Traumatic spinal cord injury (SCI) is a common traumatic disease worldwide, including permanent disability of the motor, sensory and autonomic nervous system [1]. The global incidence rate is 10.4 to 83 cases per million people per year, representing an enormous economic burden for society and families [2]. For many years, the repair of SCI has been considered a global medical challenge. The pathophysiological process is mainly divided into primary and secondary injuries [3]. As primary injury is difficult to prevent, interventions for SCI mainly focus on how to effectively reduce secondary injury. Secondary injury refers to a process of active regulation at the cellular and molecular level caused by a series of biochemical mechanisms after injury [1,4], which cause the intact tissue around the lesion to develop destructive lesions, further deepening the degree of injury and expanding the area of damage. There is increasing evidence that effectively improving the spinal cord regeneration micro-environment and increasing the number and survival rate of neurons in the injured area have become important interventions for nerve repair after SCI [5,6,7].

Neural tissues have extremely high metabolic demands. Severe metabolic damage is evident in the injured tissue following SCI, exacerbating axonal degeneration and neuronal death [8]. Nicotinamide adenine dinucleotide (NAD^+^) is an important co-factor for metabolic energy and a substrate for a wide range of enzymes [9], and plays a key role in the regulation of virtually all major biological processes [10]. Multiple pathways are involved in the synthesis and catabolism of NAD^+^ [11,12,13], and alterations in NAD^+^ homeostasis have emerged as a common feature of a wide range of disease states [14]. NAD^+^ has a role in calcium regulation, and in mitochondrial and immune functions [15]. Exogenous administration of NAD^+^ reduces oxidative stress-induced neuronal apoptosis to protect against ischemic spinal cord injury [16]. Studies have shown that NAD^+^ levels in neurons decrease after axonal injury or neurodegenerative diseases [17,18], suggesting that NAD^+^ plays a key role in the underlying process of axonal degeneration. Therefore, effectively increasing/maintaining NAD^+^ levels may become an important strategy for the treatment of SCI.

Nicotinamide riboside (NR) is a new NAD^+^ precursor found in milk that restores the bioavailability of NAD^+^ in vivo [19]. When NR enters the cell, it is catalyzed by nicotinamide riboside kinases (NRKs) and metabolized directly to nicotinamide mononucleotide (NMN), which increases the body’s NAD^+^ levels [20]. Alternatively, NR can be converted to NAM via purine nucleoside phosphorylase (NP) and then to NAD^+^ via NMNAT and NMN [12,20]. Numerous beneficial effects of NR have been reported in neurological disorders. For instance, NR has been shown to increase NAD^+^, reduce DNA damage, ameliorate neuroinflammation, attenuate cellular apoptosis, and improve hippocampal synaptic plasticity in diabetic mice and mouse models of Alzheimer’s disease [21,22]. The administration of NR increased NAD^+^ levels and significantly suppressed inflammation in the brain [23,24]. Encouragingly, Mariajose reported beneficial effects of NR after SCI in rats, and supplementation of NR to increase NAD^+^ protected spinal cord tissues from injury and promoted motor recovery [25]. However, the role of NR in mice after SCI is not fully understood and the specific mechanisms remain to be further explored.

Here, we performed in vitro and in vivo studies to elucidate the effects of NR supplementation in mice after SCI. Firstly, we confirmed the effect of NR supplementation on increasing NAD^+^ levels in mice after SCI and improving the behavioral functions of SCI mice. We also examined the effect of NR supplementation on the survival of neurons in the injured area using immunohistochemistry. Transcriptome sequencing was performed to uncover the potential mechanism. Additionally, we studied and discussed the impact effect of NR supplementation on the micro-environment after SCI in mice by semiquantitative cytokine array and qRT-PCR. Furthermore, in vitro experiments validated the effect of NR supplementation on neuronal survival under oxidative stress and inflammation models. Our results showed that NR supplementation might be an important strategy for the treatment of SCI.

## 2. Materials and Methods

### 2.1. Animals

Sixty-three adult female C57BL/6N (6–8 weeks old, 18–22 g) mice were purchased from Beijing Vital River Laboratory Animal Technology Co., Ltd. (Beijing, China). Mice were kept under standard conditions (temperature, 22 ± 2 °C; humidity, 55 ± 10%) with a 12:12 light/dark cycle. Food and water were available ad libitum. All animal protocols are approved and strictly follow the regulations of the Experimental Animal Center of Capital Medical University and the Beijing Experimental Animal Association (ethical approve No. AEEI-2023-104).

### 2.2. Spinal Cord Injury

General anesthesia was initiated with isoflurane (2 vol.%) in an anesthetic chamber. During surgery, isoflurane (1.5 vol.%) was further administered via a face mask. The T10 spinal cord was exposed by laminectomy, followed by a 70-kilodyne contusion using the Infinite Horizons Impactor (Precision Systems & Instrumentation, Lexington, KY, USA). During the surgery, body temperature was maintained at 37 °C. The animals were then taken out of anesthesia and given 0.5 mL isotonic saline and antibiotic treatment via subcutaneous injection. Bladder evacuation was manually applied twice daily until the mice could urinate spontaneously.

### 2.3. Experimental Protocol

The mice were randomly divided into four groups: (1) non-SCI without NR (sham), (2) non-SCI with NR (sham + NR), (3) SCI without NR (SCI), and (4) SCI with NR (SCI + NR). The sham group underwent laminectomy without contusion of the spinal cord. The SCI group received laminectomy with a 70-kilodyne contusion. Mice in the SCI + NR group underwent the same surgical procedure as those in the SCI group and received NR intraperitoneal injection immediately after surgery for 7 consecutive days. The sham + NR group was also established by only NR intraperitoneal injection immediately after laminectomy for 7 consecutive days without contusion.

### 2.4. NR Preparation and Treatment

NR (HY-123033A, Med Chem Express, New Jersey, NJ, USA) was dissolved in PBS solution and injected intraperitoneally. To investigate whether NR has protective effects in SCI, mice were treated with 500 mg/kg intraperitoneally [25,26,27]. The sham + NR group was treated with equivalent volume of NR intraperitoneally. The sham and SCI groups were treated with equivalent volume of PBS solution.

### 2.5. Tissue Preparation

To obtain samples for molecular biology and transcriptome sequencing, mice were deeply anesthetized and transcardially perfused with ice cold 0.9% isotonic saline solution, the epicenter part of spinal cord was quickly dissected, snap-frozen in liquid nitrogen, and stored at −80 °C until further experiments. For immunohistochemistry, mice were transcardially perfused with ice cold 0.9% isotonic saline solution followed by a 4% paraformaldehyde solution (PFA, pH 7.4) at 8 weeks after SCI. Tissue specimens were embedded in paraffin (Leica, Wetzlar, Germany) and 5 µm paraffin sections were cut.

### 2.6. Determination of NAD^+^ Content in Spinal cord Tissue

NAD^+^ levels were measured using a NAD/NADH Assay kit (Cat# ab65348,Abcam, Cambridge, UK). All procedures were conducted strictly according to the manufacturers’ instructions. Approximately 20 mg of spinal cord tissue was digested in 400 μL NADH/NAD Extraction Buffer. After centrifuging in a 10 kD Spin Column (Cat# ab93349, Abcam) at 10,000× *g* for 10 min at 4 °C, half of the sample was transferred to a new tube and incubated at 60 °C for 30 min to decompose NAD^+^, while the remaining half was used as NADtotal (NADH plus NAD^+^). 20 μL of the NADtotal and 20 μL of the decomposed NAD^+^ sample were mixed with 30 μL Extraction Buffer and then incubated with 100 μL of Reaction Mix at RT for 5 min to convert NAD^+^ to NADH. After adding 10 μL of NADH Developer into each well, it was mixed. The reaction was allowed to cycle at room temperature for 20 min. The sample outputs were measured at OD 450 nm on a microplate reader in a kinetic mode.

### 2.7. Hematoxylin-Eosin (HE) Staining

For HE staining, the sections were deparaffinized and rehydrated. After staining with hematoxylin for 1 min, the sections were washed three times in double distilled water. Then, the sections were incubated in the 1% hydrochloric alcohol differentiation for 30 s, stained with eosin for 50 s, followed by 75% ethanol, 80% ethanol, 95% ethanol, 100% ethanol, and finally cleared in xylene, and mounted by neutral resins. The image was analyzed by light microscope (Tissue Gnostics, Vienna, Austria) at 20× magnification, and the injured area was measured using three sections per mouse by Image J (version 1.53e; National Institutes of Health, Bethesda, MD, USA).

### 2.8. Immunohistochemistry

For immunohistochemistry, sections were deparaffinized, rehydrated, and antigens were unmasked by heating in Tris/EDTA (pH 9.0) buffer for 20 min. After blocking with 5% normal goat serum and 0.3% Triton X-100 in PBS, the sections were incubated overnight at 4 °C with the primary antibody diluted in blocking solution. Primary antibodies and dilutions used in the study are given in Table 1. The sections were then incubated at room temperature for 2 h with fluorescent-labeled secondary antibodies and washed with PBS before being observed under a Tissue FAXS system (Tissue Gnostics, Austria).

### 2.9. RNA Sequencing

A total amount of 1–3 μg RNA per sample was used as input material for the RNA sample preparations. Sequencing libraries were generated using VAHTS Universal V6 RNA-seq Library Prep Kit for Illumina^®^ (NR604-01/02) following the manufacturer’s recommendations and index codes were added to attribute sequences to each sample. Briefly, mRNA was purified from total RNA using poly-T oligo-attached magnetic beads. Then, we added fragmentation buffer to break the mRNA into short fragments. First strand cDNA was synthesized using random hexamer primer and RNase H. Second strand cDNA synthesis was subsequently performed using buffer, dNTPs, DNA polymerase I and RNase H. And then, the double stranded cDNA was purified by AMPure P beads or QiaQuick PCR kit. The purified double stranded cDNA was repaired at the end, adding a tail and connected to the sequencing connector, then the fragment size was selected, and finally the final cDNA library was obtained using PCR enrichment. The clustering of the index-coded samples was performed on a cBot cluster generation system using HiSeq PE Cluster Kit v4-cBot-HS (Illumina) according to the manufacturer’s instructions. After cluster generation, the libraries were sequenced on an Illumina platform and 150 bp paired-end reads were generated. The cluster generation and sequencing were performed on Novaseq 6000 S4 platform, using NovaSeq 6000 S4 Reagent kit V1.5. Transcriptome data analysis was performed via R language (4.2.3). Specifically, differential genes (DEGs) with |log_2_(fold change)| > 0.5 and *p*-value < 0.05 were identified by DESeq2 (1.38.3), and by ggplot2 (3.4.1) and pheatmap (1.0.12) Display. Gene Ontology (GO) is completed through the R package ClusterProfiler (4.7.1).

### 2.10. Semiquantitative Cytokine Array

The cytokines in mouse spinal cord were measured with a Mouse Cytokine Array GS4000 (Cat# GSM-CAA-4000, Ray Biotech, Atlanta, GA, USA) according to the manufacturer’s instructions. Protein extraction of spinal cord tissue, and the protein concentration of the samples were calculated based on the standard curve. The slide chip was removed and equilibrated to room temperature for 20–30 min and dried in a vacuum desiccator or at room temperature for 1–2 h. A sample diluent of 100 μL was added to each well and incubated in a shaker at room temperature for 1h to seal the quantitative antibody chip. The buffer was removed from each hole, a 100 μL sample was added to the hole (diluted at 500 μg/mL), and incubated overnight at 4 °C on a shaker. Then, the Wellwash Versa chip washer (Thermo Scientific, Waltham, MA, USA) was ued to clean the slides. The antibody mixture tubules were centrifuged, then 1.4 mL of sample diluent was added, and centrifuged again quickly. A total of 80 μL of detection antibody was added to each well and incubated in a room temperature shaker for 2 h. The slides were washed as described above. A total of 80 µL of Cy3-streptavidin was added to each well, and incubated away from light at room temperature for 1 h, and washed again. The fluorescein-labeled array was visualized using an InnoScan 300 Microarray Scanner. Data were extracted by GenePix Pro 5.1 software and analyzed with RayBiotech Q-Analyzer software (SA52).

### 2.11. Quantitative Real-Time PCR (qRT-PCR) Analysis

Total RNA and DNA were isolated using Trizol reagent (Invitrogen, Carlsbad, CA, USA) and a DNeasy Tissue Kit (Qiagen, Valencia, CA, USA), respectively. All primers used are shown in Table 2. The mRNA expression levels of genes were detected. For the qPCR of mRNA expression levels of genes, reverse transcription was carried out, followed by real-time PCR amplification. mRNA expression levels were normalized against reference gene GAPDH and measured using the ∆∆CT method.

### 2.12. Cell Cultures and Treatment

The HT-22 cell lines were obtained from Biological Medicine Cell Resource (BMCR, Beijing, China). The cell lines were cultured in Dulbecco’s Modified Eagle’s Medium (DMEM, Gibco, New York, NY, USA) supplemented with 10% foetal bovine serum (FBS, Gibco), penicillin and streptomycin (100 U/mL, Gibco, Waltham, MA, USA) in a humidified incubator with 5% CO_2_ at 37 °C. The cells were pretreated with NR (0.5 μM) for 1 h followed by post-incubation with LPS (1 μg/mL) or H_2_O_2_ (100 μM) for 24 h. The concentration of NR was selected by the CKK8 assay, and the concentration of LPS and H_2_O_2_ was considered according to previously described studies [28,29].

### 2.13. CCK8 Assay

Cell viability was analyzed by Cell Counting Kit-8 (CCK8, Beyotime, Shanghai, China) according to the manufacturer’s protocols. Cells were seeded and cultured at a density of 5 × 10^3^/well in 100 μL of medium into 96-well microplates (Corning, New York, NY, USA). Then, the cells were treated with various treatments. After 24 h, 10 μL of CCK-8 reagent was added to each well and then cultured for 2 h. All experiments were performed in triplicate. The absorbance was analyzed at 450 nm using a microplate reader (PerkinElmer, EnSpire, Waltham, MA, USA) using wells without cells as blanks. The proliferation of cells was expressed by the absorbance.

### 2.14. Behavior Evaluation

#### 2.14.1. Basso Mouse Scale

Hind limb locomotor function in an open field was assessed with the Basso Mouse Scale (BMS) [30], using a 0 to 9-point scale (complete paralysis to normal hind limb function), by two experienced investigators who were blinded to experimental treatment and observed open-field locomotion for over 4 min on days 1, 3, and 7 post injury, then once weekly thereafter for next 7 weeks.

#### 2.14.2. Grip Strength Test

A grip strength meter (Chatillon force measurement, Ametek, New York, NY, USA) was used to assess the forelimb and hindlimb grip strength of the mice. Mice were lifted by the tail and induced to grasp a rigid grid attached to a digital force gauge. The tail of each mouse was gently pulled backwards and the tension reading of the digital force gauge was defined as the grip strength before the mouse released the net. Five consecutive tests were performed on each mouse and the mean maximum limb muscle strength value (grams) (g) was calculated.

#### 2.14.3. Open Field Test

To further assess the locomotion capabilities of the mice, we conducted open field tests using TopScan software (version 2.00, Clever Systems, Reston, VA, USA). It was performed in this study based on our previous descriptions [31]. Briefly, mice were exposed to an open arena (50 cm × 50 cm × 50 cm, length × width × height) under dimmed lighting (20 lx). The inner wall and bottom surface of the open field test box were cleaned with 70% ethanol. A mounted camera was used to record each trial and analyzed the total distance travelled in the open arena for 5 min.

### 2.15. Statistical Analysis

The data were analyzed by GraphPad Prim (8.0). All values are presented as the mean ± standard error of the mean (SEM). Two groups of data were analyzed by Student’s *t* tests. One-way ANOVA with Tukey’s multiple comparisons posttest or two-way ANOVA-RM with Bonferroni’s post hoc correction were used when comparing multiple groups. A *p* value of less than 0.05 was considered statistically significant.

## 3. Results

### 3.1. NR Supplementation Significantly Improved Motor Function in Mice after SCI

**Figure 1:**
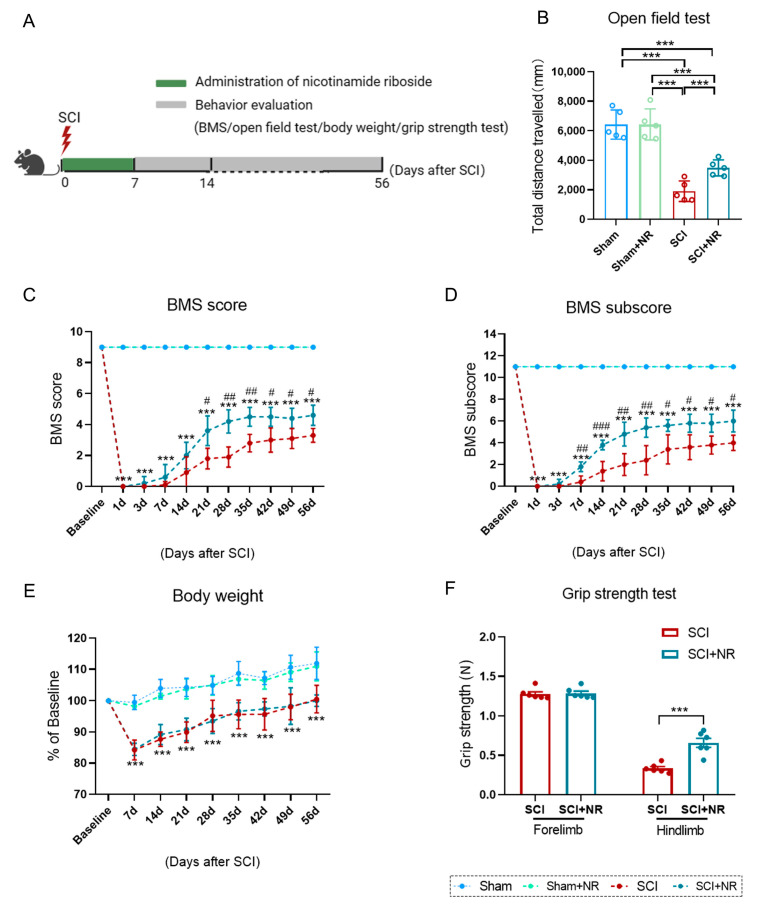
NR supplementation promoted recovery of motor function in mice after SCI. (**A**) Schematic representation of the experiments. (**B**) Distance traveled during the open-field test. (**C**,**D**) Motor function score over time after SCI as assessed by BMS. (**E**) Changes in body weight over time in the four groups. (**F**) Experimental animal grip strength assessment of the SCI groups and SCI + NR groups at 56 days after SCI (*** *p* < 0.001 compared with the sham group; # *p* < 0.05, ## *p* < 0.01, and ### *p* < 0.001 compared with the SCI group, *n* = 5 mice/group).

To investigate the role of NR in vivo, we used a contusive SCI mouse model. After contusive SCI in mice, NR was administered via intraperitoneal administration at 500 mg/kg for 1 week (Figure 1A). An open field test was used to measure spontaneous locomotor activity at 56 days post-injury. The data showed that the total distance traveled between the SCI group and SCI + NR group were significantly different (Figure 1B). Commencing at three weeks post-injury and persisting throughout the experimental period, NR treatment exhibited notable efficacy in enhancing hindlimb behavior (as measured by BMS main scores) in SCI mice (Figure 1C). Specifically, a significant improvement in BMS sub-scores was observed at one week post-injury in comparison to the SCI group, and this improvement was sustained throughout the post-injury period (Figure 1D). Importantly, the administration of NR at a safe dosage had minimal impact on the body weight of mice following SCI (Figure 1E). At 8 weeks postoperatively, treatment with NR demonstrated a significant enhancement in the grip strength of the hind limbs in mice with SCI, while no significant effect was observed on the grip strength of the forelimbs (Figure 1F). These results indicated that the supplementation of NR significantly improved the motor function of the hind limbs in mice after SCI.

### 3.2. Supplementation of NR Significantly Increased the Level of NAD^+^ in the Injured Spinal Cord of SCI Mice and Promoted Cell Survival

**Figure 2:**
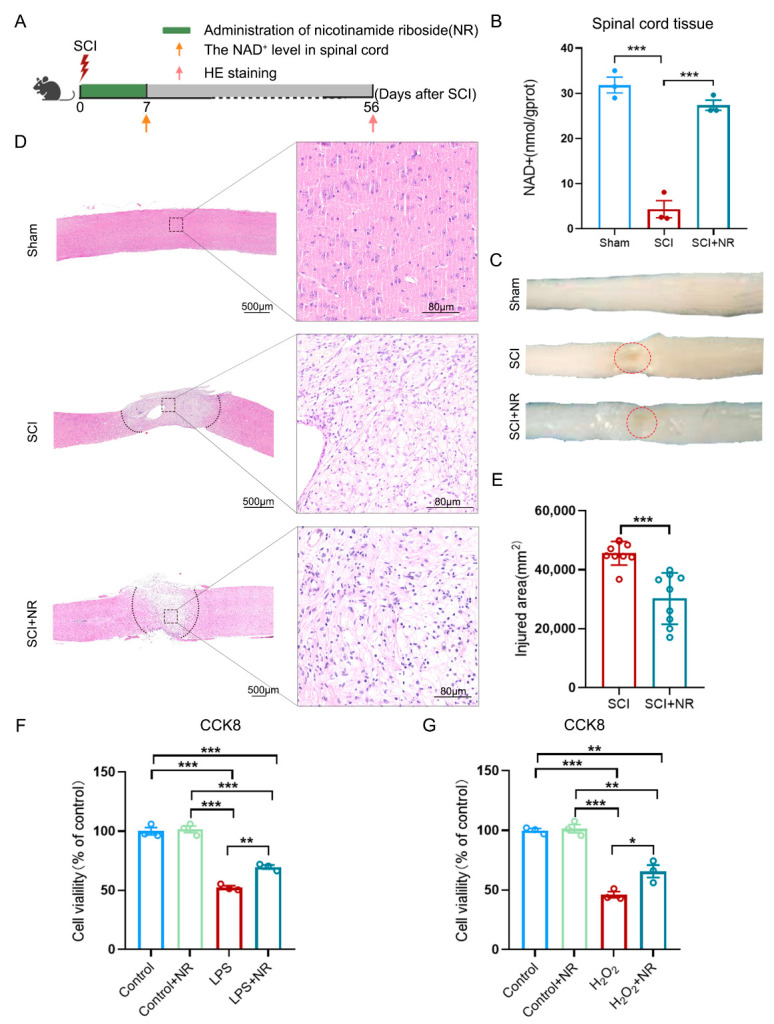
NR supplementation elevated the NAD^+^ level in spinal cord tissue after SCI and promoted cell survival. (**A**) Schematic representation of the experimental procedure in vivo. (**B**) The level of NAD^+^ in spinal cord tissue of each group after SCI. (**C**) Organizational visualization of each group after SCI. The red dotted circles show the injured area. (**D**,**E**) Representative images of hematoxylin-eosin (HE) staining of spinal cord and quantitative analysis of injured area between the groups of SCI and SCI + NR. The black solid dots indicate the boundary between the host spinal cord and the injured area. (**F**) Quantitative analysis of cell viability in each group after LPS stimulation. (**G**) Quantitative analysis of cell viability in each group after H_2_O_2_ stimulation. One-way ANOVA and Tukey’s post hoc test for multiple comparisons; * *p* < 0.05, ** *p* < 0.01, *** *p* < 0.001; Mean ± S.E.M. (*n* = 3 mice or triple repetition/group).

NAD^+^ is an essential coenzyme for energy metabolism and plays a crucial role in various biological processes including metabolism, aging, cell death, DNA repair, and gene expression [13]. NR, as one of the NAD^+^ precursors, can be metabolized to nicotinamide mononucleotide (NMN) through the catalysis of nicotinamide ribonucleotide kinases (NRKs) and subsequently be synthesized into NAD^+^ by nicotinamide/nicotinamide mononucleotide adenylyltransferase (NMNAT) for the synthesis of NAD^+^ [12,20]. To observe the effect of NR supplementation on NAD^+^ levels in spinal cord tissues of mice after SCI, we assessed NAD^+^ levels using the NAD/NADH Assay kit 7 days after SCI (Figure 2A). The results showed a significant decrease in NAD^+^ levels in the spinal cord tissues of SCI mice compared to the sham group (Figure 2B), which was consistent with previous reports of decreased NAD^+^ levels following organismal injury [32,33]. Following the administration of NR, the decrease in NAD^+^ levels within the spinal cord tissue of SCI mice was reversed and could be significantly increased by nearly 5-fold (Figure 2B). Figure 2C showed the formation of a localized cavity in the SCI lesion 56 days after SCI, while the administration of NR reduced the damage range at the lesion site and the extent of the injury. Hematoxylin-eosin (HE) staining revealed that the extent of damage at the injury site in SCI mice administered with NR was lower than that in the SCI group mice (Figure 2D,E). In this study, HT22 cells were stimulated with LPS to simulate an in vitro inflammation model. The administration of NR significantly rescued the inflammation-induced decrease in neuronal cell viability, with HT22 cell survival rates increasing from 52.48% to 69.72% (Figure 2F). Additionally, we stimulated HT22 cells with H_2_O_2_ to simulate oxidative stress in vitro. Subsequently, NR was administered, which rescued the oxidative stress-induced decrease in neuronal cell viability. As a result, HT22 cell viability increased from 46.04% to 65.67% (Figure 2G). These results confirmed that NR supplementation promoted neuronal survival after injury. The above data demonstrated that NR supplementation protected injured spinal cord tissue and promoted cell survival by increasing NAD^+^ levels, both in vitro and in vivo.

### 3.3. Supplementing NR Promoted Neuronal Survival and Axonal Growth in the Injured Area of Mice after SCI

**Figure 3:**
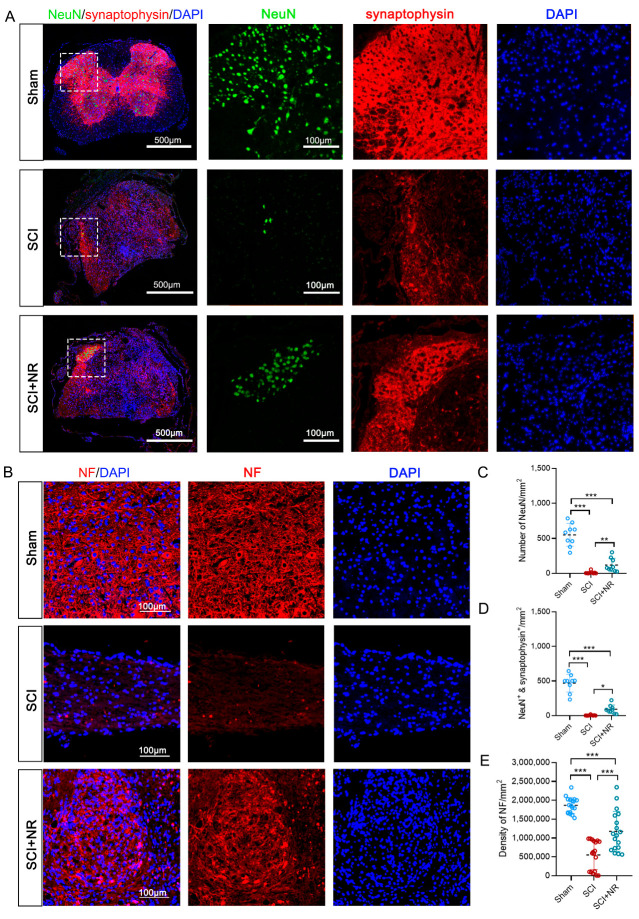
NR supplementation promoted neuronal survival and axonal growth in the injured area of mice after SCI. (**A**) Immunofluorescent staining of NeuN (green), synaptophysin (red), and DAPI (blue). Scale bars, 500 μm and 100 μm. (**B**) Representation images of axons immunostained with NF (red) and DAPI (blue) in the injured epicenter or corresponding location. Scale bars, 100 μm. (**C**) and (**D**) Quantification of the number of NeuN and NeuN/synaptophysin of each group in injured area. (**E**) Quantification of the density of NF of each group in the injured area. Data was presented as Mean ± S.E.M. * *p* < 0.05; ** *p* < 0.01; *** *p* < 0.001; *n* = 3 mice/group.

NR has been shown to be a superior neuroprotective agent to NAD^+^ in excitotoxicity-induced axonal degeneration [34] and can effectively promote neuronal survival [35]. Synapses are fundamental to neuronal activity [36]. We investigated the effects of NR supplementation on neuronal and synaptic survival in the injured area after SCI by means of NeuN/synaptophysin. The number of neurons (NeuN) and synapses (synaptophysin) in the injured area were significantly decreased after SCI. Compared with the SCI group, NR supplementation significantly increased the number of NeuN+ neurons in the injured area (Figure 3A,C). Additionally, the amount of NeuN/synaptophysin double positivity was significantly increased (Figure 3A,E), suggesting that NR supplementation promoted the survival of neurons and synapses in the injured area of mice after SCI. To observe the effect of NR supplementation on axonal regeneration in mice after SCI, we quantified the fluorescence density of NF by immunofluorescence staining. The results confirmed that NR supplementation significantly promoted axonal regeneration in the injury area compared with the SCI group (Figure 3B,E). The above results suggested that NR supplementation promoted neuronal survival and axonal growth in the injured area of mice after SCI. These data together suggested that NR supplementation promoted neuronal survival and axonal growth in the injured area of mice after SCI.

### 3.4. Supplementation of NR Regulates Chemotaxis in Early SCI

**Figure 4:**
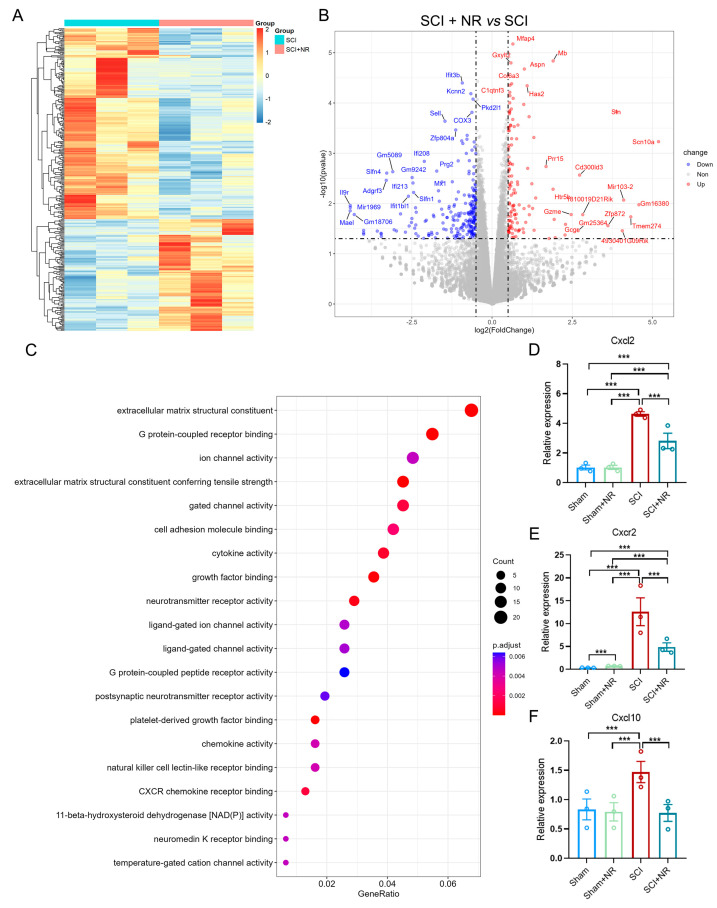
Supplemental NR downregulated key chemotactic signaling pathways in the early stage of SCI. (**A**) Significant differential expression analysis (DEGs) (*p* < 0.05) after RNA-seq analysis. (**B**) DEGs in the SCI + NR group versus the SCI group shown in the volcano plot. (**C**) GO terms: molecular function changed in injured spinal cord after NR treatment. (**D**–**F**) Relative mRNA levels of Cxcl2, Cxcr2 and Cxcl10 by quantitative real-time PCR. Data was presented as Mean ± S.E.M. *** *p* < 0.0001, *n* = 3 mice/group.

To investigate the mechanism by which NR supplementation exerts its beneficial effects after SCI, we performed RNA-seq analyses of spinal cord tissues in the injured area of each group following one week of NR administration. As shown in the heat and volcano plots, 342 DEGs were identified in the injured spinal cord tissues of SCI mice after NR treatment, as compared to the SCI group (Figure 4A,B). The GO enrichment analysis results indicated enrichment for various processes, including cell adhesion molecule binding, cytokine activity, growth factor binding, neurotransmitter receptor activity, postsynaptic neurotransmitter receptor activity, chemokine activity, CXCR chemokine receptor binding, and NAD(P) activity, among other important processes (Figure 4C). In the GO enrichment analysis above, we focused on the CXCR chemokine receptor binding process. The gene expression of Cxcl2, Cxcr2, and Cxcl10 was significantly down-regulated in the spinal cord lesion area of SCI mice after NR supplementation compared to the SCI group. The up-regulation of Cxcl2, Cxcr2, and Cxcl10 mRNA expression after SCI was further verified by qRT-PCR. The expression of these three chemokines was significantly down-regulated by NR supplementation (Figure 4D–F). The results indicated that NR supplementation might have a protective effect on the focal spinal cord of SCI mice by reducing the early chemotaxis effect of SCI.

### 3.5. NR Supplementation Attenuates the Immune Inflammatory Response after SCI

**Figure 5:**
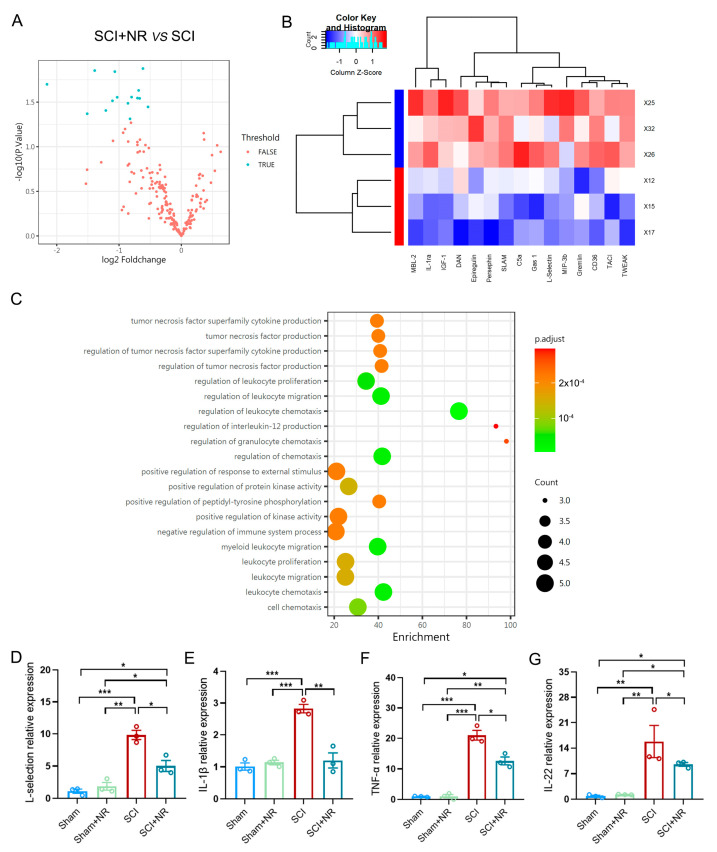
NR supplementation significantly reduced the inflammatory response after SCI. (**A**) Volcano plot of differential genes between SCI group and SCI + NR group. (**B**) Heat map of differential genes between SCI group and SCI + NR group (SCI group: X25/X32/X26; SCI + NR group: X12/X15/X17). (**C**) Associated GO terms: biological process changed in injured spinal cord after NR treatment. (**D**) Relative mRNA levels of L-selection normalized to GAPDH transcript in each group of injured spinal cord. (**E**–**G**) Relative mRNA levels of IL-1β (**E**), TNF-α (**F**) and IL-22 (**G**) normalized to GAPDH transcript in each group of injured spinal cord. Data was presented as Mean ± S.E.M. * *p* < 0.05, ** *p* < 0.01, *** *p* < 0.0001, *n* = 3/group.

The anti-inflammatory effect of NR in AD mouse models [37] and oral NR supplementation in the elderly increased the NAD^+^ metabolome of skeletal muscle and induced anti-inflammatory characteristics [38], which led us to notice the potential of NR improving the immune micro-environment to the injured area after SCI. To investigate the potential of NR supplementation in improving the immune micro-environment through chemotaxis in SCI mice, we conducted a study using Mouse Cytokine Array GS4000. We analyzed the changes in cytokines in the injured area of mice from each group 7 days after SCI. The results from the volcano plot indicated that there were 15 differential proteins in the SCI + NR group compared to the SCI group (Figure 5A). Notably, NR supplementation significantly down-regulates the expression of C5a, L-selection, CD36, TACI, and other proteins (Figure 5A,B). Consistent with the RNA-seq results, the GO terms: biological process revealed enrichment in the regulation of chemotaxis, lymphocyte proliferation, and migration (Figure 5C). This suggested that NR supplementation might be involved in regulating immune cell proliferation and chemotaxis. L-selection, a major regulator of leukocyte adhesion, migration, and signal transduction [39], was found to be up-regulated after SCI (Figure 5D). However, it was down-regulated by NR supplementation, as confirmed by qRT-PCR (Figure 5D). Additionally, we investigated the changes in inflammation-related cytokines in the injured area using qRT-PCR. The expression of IL-1β, TNF-α, and IL-22 in the injured area of mice in the SCI group was significantly increased compared to the sham group. NR supplementation led to a significant down-regulation of the relative expression of IL-1β, TNF-α, and IL-22 in SCI mice (Figure 5E–G). These findings suggest that NR supplementation might reduce the chemotaxis/recruitment of immune cells and attenuate the local immune inflammatory response in the injured area after SCI.

## 4. Discussion

In this study, we revealed a new mechanism by which NR supplementation exerts anti-inflammatory effects by attenuating leukocyte chemotaxis after SCI, thereby promoting neuroprotection. We confirmed that the administration of NR to SCI mice could effectively increase NAD^+^ levels in spinal cord tissue and promote the recovery of motor function. Through in vivo and in vitro evidence, we showed that NR could promote the survival of neurons after injury, increase the number of synapses, and regenerate axons. This protective effect might be exerted through the mechanism of regulating the local immune micro-environment of SCI by reducing leukocytes chemotaxis/recruitment in the early stages of SCI. Our data showed for the first time that NR supplementation exerted anti-inflammatory effects in mice after SCI in a manner that alleviated leukocyte chemotaxis.

NR, as an effective NAD^+^ precursor, has a two-step or three-step pathway to form NAD^+^ and its precursors, which must be converted into NR or NAM before entering cells [40]. Currently, NR is emerging as a leading drug candidate compared to other precursors (NAM/NMN) due to its high bioavailability, safety, and strong ability to increase NAD^+^ levels [41,42]. Therapeutic strategies to maintain or increase NAD^+^ early after injury may reduce the progression of secondary injury and tissue damage [24,34]. This study showed that in mice after SCI, there was a depletion of NAD^+^ in the lesional spinal cord tissue. NR as a dietary supplement had been shown to safely increase NAD^+^ levels in humans [43]. To get closer to clinical research, we provided NR supplements to mice immediately after SCI for 7 days, which confirmed that it was indeed effective in increasing NAD^+^ levels in the spinal cord tissue of mice in the acute phase of SCI.

It has been confirmed in in vivo and in vitro models that NAD^+^ is crucial for energy metabolism, oxidative stress, DNA damage repair, lifespan regulation, and some signaling processes, and can prevent neurodegeneration [24] and enhance axonal protection [34]. In the spinal cord ischemia-reperfusion injury model, Xie et al. confirmed that supplementing NAD^+^ reduce oxidative stress and neuronal apoptosis [16,44]. NR is an effective precursor supplement for NAD^+^. NR supplementation has been shown multiple times to increase NAD^+^ levels and a range of its related metabolites. In mice models, NR increased NAD^+^ metabolism, which improved glucose tolerance, reduced weight gain, and exhibited neuroprotective effects against diabetic neuropathy and hepatic steatosis [45]. NR preserves cardiac function in a mouse model of dilated cardiomyopathy by stabilizing myocardial NAD^+^ levels in the failing heart [46]. Long-term NR supplementation improved muscle mitochondrial biogenesis, satellite cell differentiation, gut microbiota, and DNA methylation in humans [47]. NR protected against ethanol induced liver injuries via replenishing NAD^+^, reducing oxidative stress, and activating SirT1-PGC-1α-mitochondrial biosynthesis [48]. Although the specific mechanisms have not been thoroughly investigated, NR treatment could promote the preservation of neurons after SCI in rats [25], which is also consistent with our results. NR promoted oxidation resistance and upregulated biological pathways associated with synaptic transmission and PPAR signaling, which protect the synapse and prevent hearing loss [49,50]. Our study found that NR supplementation significantly increased the number of synapses in the spinal cord tissue of SCI lesions. Furthermore, NAD^+^ displayed significant neuroprotective properties in cultured neurons [51]. Our in vitro cell experiments also confirmed that NR could increase the survival of neuronal cells under LPS inflammatory stimulation and the H_2_O_2_ oxidative stress model. Supplementation of NAD^+^ with NR slowed axon degeneration and demyelination in a mouse facial nerve axotomy model [52]. We found that supplementing NR enhanced the protective effect of axons in mice after SCI.

NR improved neuroinflammation [37] and had anti-inflammatory effects in clinical studies [38], which provided evidence to support our research on supplementing NR to improve the immune micro-environment of the injured area after SCI. In this study, supplementing NR could down-regulate the levels of TNF-α, IL-1β, and IL-22 in the spinal cord lesion tissues of SCI mice and improve the inflammatory micro-environment. Supplementing with NR might reduce inflammatory cytokine secretion by inhibiting leukocyte proliferation and migration. Both the Mouse Cytokine Array GS4000 and qRT-PCR confirmed that NR supplementation could inhibit the expression level of L-selection in mouse lesion tissue after SCI. L-selection was a major regulator related to leukocyte adhesion, migration, and signal transduction. At the same time, both RNA-seq and Mouse Cytokine Array GS4000 results indicated that supplementing NR could regulate important biological processes related to leukocyte chemotaxis.

Studies had shown that NR could reverse the progressive atrophy syndrome of skeletal muscles in mice lacking Nampt while restoring endurance within 1 week of treatment [53]. NR significantly increased the content of NAD^+^ in muscles [41], which could effectively delay the progression of muscle atrophy and degeneration by improving muscle strength, restoring aging muscle stem cells, reducing inflammation and fibrosis levels [20]. Our results showed that supplementing NR could improve BMS scores, improve motor function, and increase the grip strength level of injured hind limbs in mice. The neuroprotective effects of NR supplementation may be related to improved neuronal survival and axon regeneration after SCI. This study provided new evidence supporting the understanding of neuroprotection and improved immune micro-environment after SCI through NR supplementation.

## 5. Conclusions

NR, as a safe precursor to increase NAD^+^ levels, could be used as an effective strategy to reduce secondary damage. Our research showed that NR treatment improved the local inflammatory micro-environment of the lesion by reducing the chemotaxis of leukocytes, enhanced tissue preservation after SCI, and ultimately promoted the recovery of motor functions. Further studies in appropriate in vivo and in vitro models are still needed to study these effects in the future. In summary, this study provided new data to explore the relationship between NAD^+^ levels and the regulation and functional recovery of the damaged immune micro-environment in mice after SCI.

## Figures and Tables

**Table 1:** List of antibodies used for immunohistochemical staining.

Antibody	Host	Dilution	Manufacturer	Catalog
NeuN	Rabbit	1:500	Abcam	ab177487
Synaptophysin	Rabbit	1:400	Abcam	ab32127
NF	Mouse	1:400	Cell Signaling Technology, Boston, MA, USA	2836s

**Table 2:** Primer sequences of qRT-PCR.

Items	Primer (5′→3′)	Primer (3′→5′)
Cxcl2	TGAACAAAGGCAAGGCTAACTGA	TAACAACATCTGGGCAATGGAAT
Cxcr2	ATGCCCTCTATTCTGCCAGAT	GTGCTCCGGTTGTATAAGATGAC
Cxcl10	CCAAGTGCTGCCGTCATTTTC	GGCTCGCAGGGATGATTTCAA
L-Selection	TACATTGCCCAAAAGCCCTTAT	CATCGTTCCATTTCCCAGAGTC
IL-22	ACATTATCTGCTATTGATATTTAGT	CATGTGTTTATTAAAGCCTAAGA
TNF-α	CCTCTTCTCATTCCTGCTTGTG	GGTCTGGGCCATAGAACTGAT
IL-1β	GCCACCTTTTGACAGTGATG	CCACAGCCACAATGAGTGATA
GAPDH	CCTCGTCCCGTAGACAAAATG	TGAGGTCAATGAAGGGGTCGT

## Data Availability

Data are available from the corresponding author upon request.
